# X-linked *FHL1* as a novel therapeutic target for head and neck squamous cell carcinoma

**DOI:** 10.18632/oncotarget.7478

**Published:** 2016-02-18

**Authors:** Wei Cao, Jiannan Liu, Ronghui Xia, Lu Lin, Xu Wang, Meng Xiao, Chenping Zhang, Jiang Li, Tong Ji, Wantao Chen

**Affiliations:** ^1^ Department of Oral Maxillofacial-Head and Neck Oncology, Ninth People's Hospital, Shanghai Jiao Tong University School of Medicine, Shanghai 200011, China; ^2^ Shanghai Research Institute of Stomatology and Shanghai Key Laboratory of Stomatology, Shanghai 200011, China; ^3^ Department of Oral Pathology, Ninth People's Hospital, Shanghai Jiao Tong University School of Medicine, Shanghai 200011, China; ^4^ Department of Medical Records, Ninth People's Hospital, Shanghai Jiao Tong University School of Medicine, Shanghai 200011, China

**Keywords:** head and neck squamous cell carcinoma, FHL1, chromosome X, tumor suppressor, prognosis

## Abstract

To identify X-linked novel tumor suppressors could provide novel insights to improve prognostic prediction and therapeutic strategy for some cancers. Using bioinformatics and Venn analysis of gene transcriptional profiling, we identified downregulation of X-linked four-and-a-half LIM domains protein 1 (*FHL1*) gene in head and neck squamous cell carcinoma (HNSCC). *FHL1* functions were investigated and confirmed *in vitro* and *in vivo*. FHL1 downregulated mechanisms were analyzed in HNSCCs by using methylation specific PCR, bisulfate-based sequencing, 5-Aza-dC treatment and chromatin immunoprecipitation assays. Two independent HNSCC cohorts (the training cohort *n* = 105 and the validation cohort *n* = 101) were enrolled to evaluate clinical implications of FHL1 expression by using real-time PCR or immunohistochemistry. *FHL1* mRNA and protein expressions were frequently decreased in HNSCCs. FHL1 overexpression or depletion gave rise to suppress or promote cell growth through Cyclin D1, Cyclin E and p27 dysregulations. Abundant occupy of EZH2 or H3K27Me3 was observed in *FHL1* promoter except for DNA hypermethylation. Reduced FHL1 mRNA expression was notably associated with poor differentiation (*p* = 0.020). Multivariate analysis demonstrated FHL1 mRNA expression was identified as independent prognostic predictors of overall survival (OS) (*p* = 0.036; HR 0.520; Cl, 0.283–0.958) and disease-free survival (DFS) (*p* = 0.041; HR 0.527; Cl, 0.284–0.975), which was validated by another independent cohort (*p* = 0.021; HR 0.404; Cl, 0.187–0.871 for OS; *p* = 0.011; HR 0.407; Cl, 0.203–0.815 for DFS). These results suggest epigenetic silencing of X-linked FHL1 may have an important role in adjuvant therapeutic intervention of HNSCCs and is an independent prognostic factor in patients with HNSCCs.

## INTRODUCTION

Head and neck cancer, squamous cell carcinoma (HNSCC) accounts for more than 90%, is widely represented as a heterogeneous solid tumor with more aggressive behaviors [1]. Despite ongoing efforts over the past several decades, radical surgery combined with radiotherapy and chemotherapy has not notably improved the 5-year survival rate of patients with HNSCC. The mainly reason contributing to the worse survival is because of the absence of robust therapeutic target in HNSCC development. It is well known that the development of HNSCC is a multi-step process in which the activation of oncogenes and inactivation of tumor suppressor genes, such as mutations of *TP53* and *CDKN2A* and amplification of *Cyclin D1* and *EGFR* [2–4]. Accordingly, better understanding the molecular basis of HNSCCs could further facilitate development of novel strategies to improve treatment of HNSCCs.

In mammals, X chromosome is unique, because both male and female cells carry only one active X chromosome. Tumor suppressor genes (TSGs) on chromosome X as ‘high risky’ genes can be inactivated by a single hit [5]. So far, several famous X-linked TSGs have been identified involving in cancer development, such as *FOXP3* in glandular epithelial cancers [6], *WTX* in Wilms tumors [7], *USP9X* in pancreatic ductal adenocarcinomas [8] and *RSK4* in endometrial cancers [9].

To identify X-linked TSGs in HNSCCs, a bioinformatics and Venn analysis of published transcriptional profiling were performed in this study. We identified the four-and-a-half LIM domains protein 1 (*FHL1*) gene from the 48 genes on chromosome X, which expression was over 2-fold differentially expressed in HNSCCs compared to normal adjacent tissues. Our findings further demonstrate that the downregulation of *FHL1* in HNSCCs is commonly caused by hypermethylation on DNA promoter regions and EZH2-mediated histone methylation regulation. Silencing of *FHL1* notably enhanced proliferation potential of HNSCC cells, whereas forced expression of *FHL1* expression dramatically repressed growth of HNSCC cells *in vitro* and *in vivo*. High FHL1 transcriptional and translational levels were significantly associated with well differentiation, overall survival (OS) and disease-free survival (DFS) of patients with HNSCCs.

## RESULTS

### Screening for X-Linked candidate TSGs in HNSCC

**Figure 1:**
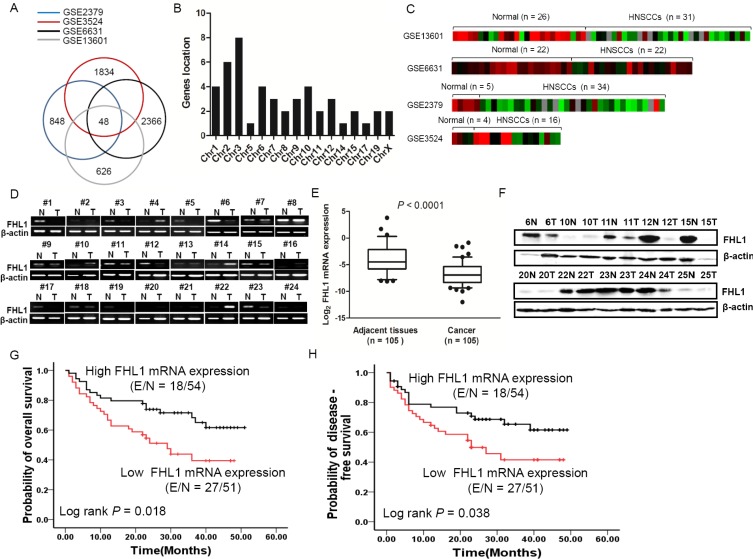
Downregulation of FHL1 was identified in four GSE databases (GSE2379, GSE3524, GSE6631 and GSE13601) and 105 HNSCCs (**A**) 48 differentially expressed genes were identified by bioinformatics and Venn analysis from four GSE databases (blue cycle indicates GSE2379, red cycle indicates GSE3524, black cycle indicates GSE6631 and gay cycle indicates GSE13601). (**B**) 48 differentially expressed genes were localized in diverse Chromosomes. (**C**) The Heatmap of X-linked *FHL1* was shown in four GSE databases. (**D**) Expression level of *FHL1* mRNA in 24 representative HNSCCs and the corresponding adjacent tissues by using RT-PCR. (**E**) Expression levels of *FHL1* mRNA were decreased in HNSCCs (*n* = 105) compared with adjacent tissues (*n* = 69) using real-time PCR analysis. For each sample, the relative mRNA level of *FHL1* was normalized to β-action. The line within each box represents the median negative Ct value; the upper and lower edges of each box represent the 75th and 25th percentiles, respectively. (**F**) Expression level of FHL1 in 10 representative HNSCCs and the corresponding adjacent tissues tested by Western blotting. (**G**) Kaplan-Meier survival curve indicated overall survival by evaluation of *FHL1* mRNA levels in the training cohort. (**H**) Kaplan-Meier survival curve indicated disease-free survival by evaluation of *FHL1* mRNA levels in the training cohort.

For a common characteristics of TSGs in human cancer is aberrant silencing, we initially screened for potential TSGs by determining transcripts that were downregulated in HNSCCs in four data sets (GSE2379, GSE6631, GSE3524, and GSE13601) (Figure 1A). A Venn analysis further yielded 48 differentially expressed genes (≥ 2 fold) in those data sets with a FDR value < 0.05 (Supplementary Table S1). Using gene location distribution (Figure 1B), *FHL1* (chrXq26) was identified with continuous decreased expression in four data sets (Figure 1C).

We next examined the FHL1 expression patterns in HNSCC tissues. FHL*1* mRNA expression was validated in HNSCC samples (*n* = 105) and paired ANTs (*n* = 69) by real-time PCR, RT-PCR and Western blotting. Our findings indicated that *FHL1* mRNA levels and protein levels were notably reduced in HNSCCs as compared with PNA (Figure 1D and 1F).

### FHL1 expression patterns were associated with poorer differentiation and worse clinical outcome

**Figure 2:**
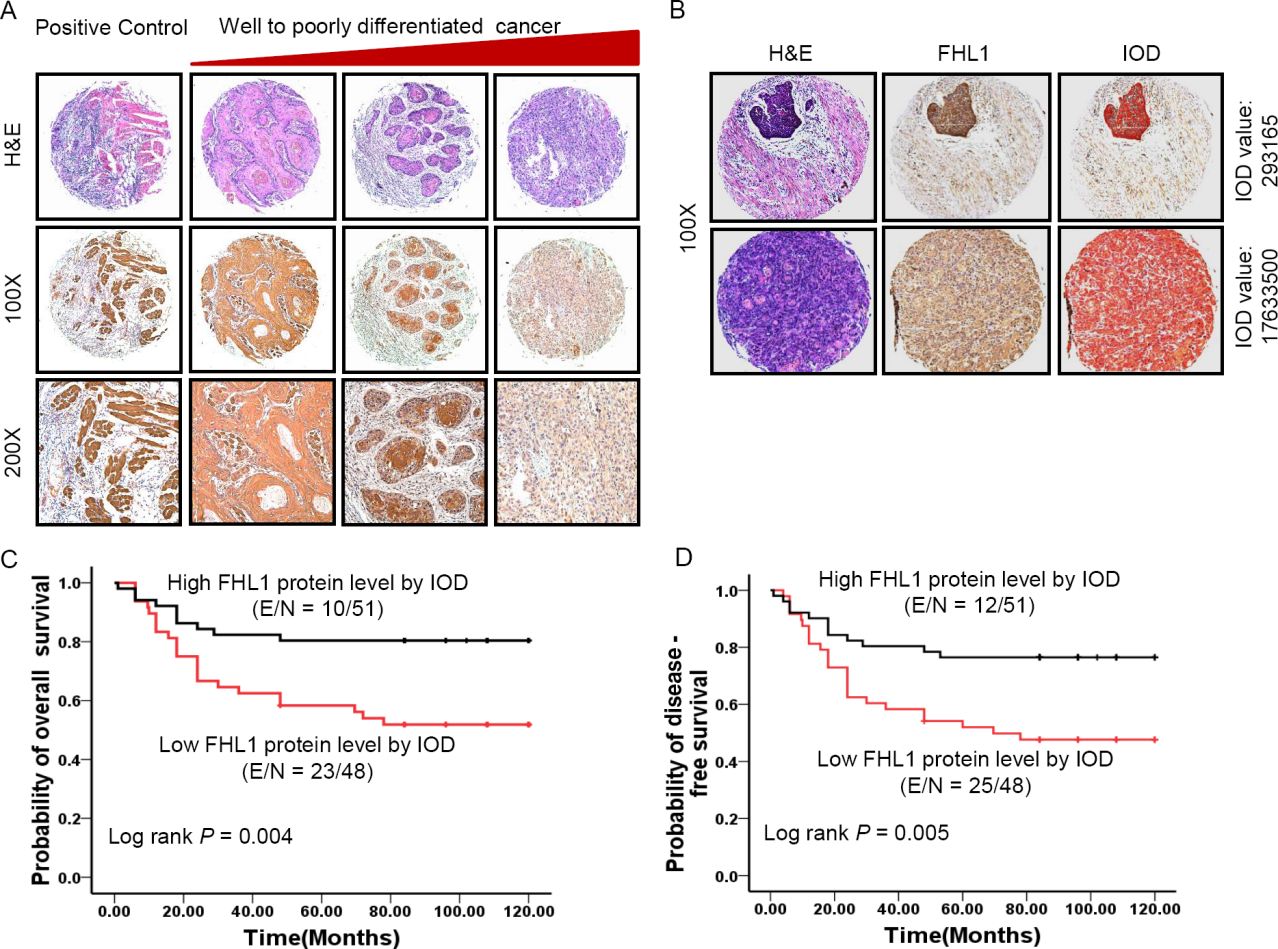
Patterns of FHL1 gene expression were investigated in 101 specimens of head and neck squamous cell carcinoma (HNSCC) (**A**) Representative images show immunohistochemical staining (IHC) for FHL1 expression in positive control (muscle), well differentiated HNSCC, moderately differentiated HNSCC, and poorly differentiated HNSCC from the left side to the right side. From the first line to the end line indicates H & E staining, ×100 original magnification and ×200 original magnification, respectively. (**B**) Representative images showed FHL1 expression was quantified by using digital image analysis (IOD value). (**C**) Kaplan-Meier survival curve indicated overall survival by evaluation of FHL1 protein levels in the validation cohort through human semiquantitative analysis. (**D**) Kaplan-Meier survival curve indicated disease-free survival by evaluation of FHL1 protein levels in the validation cohort through human semiquantitative analysis.

**Figure 3:**
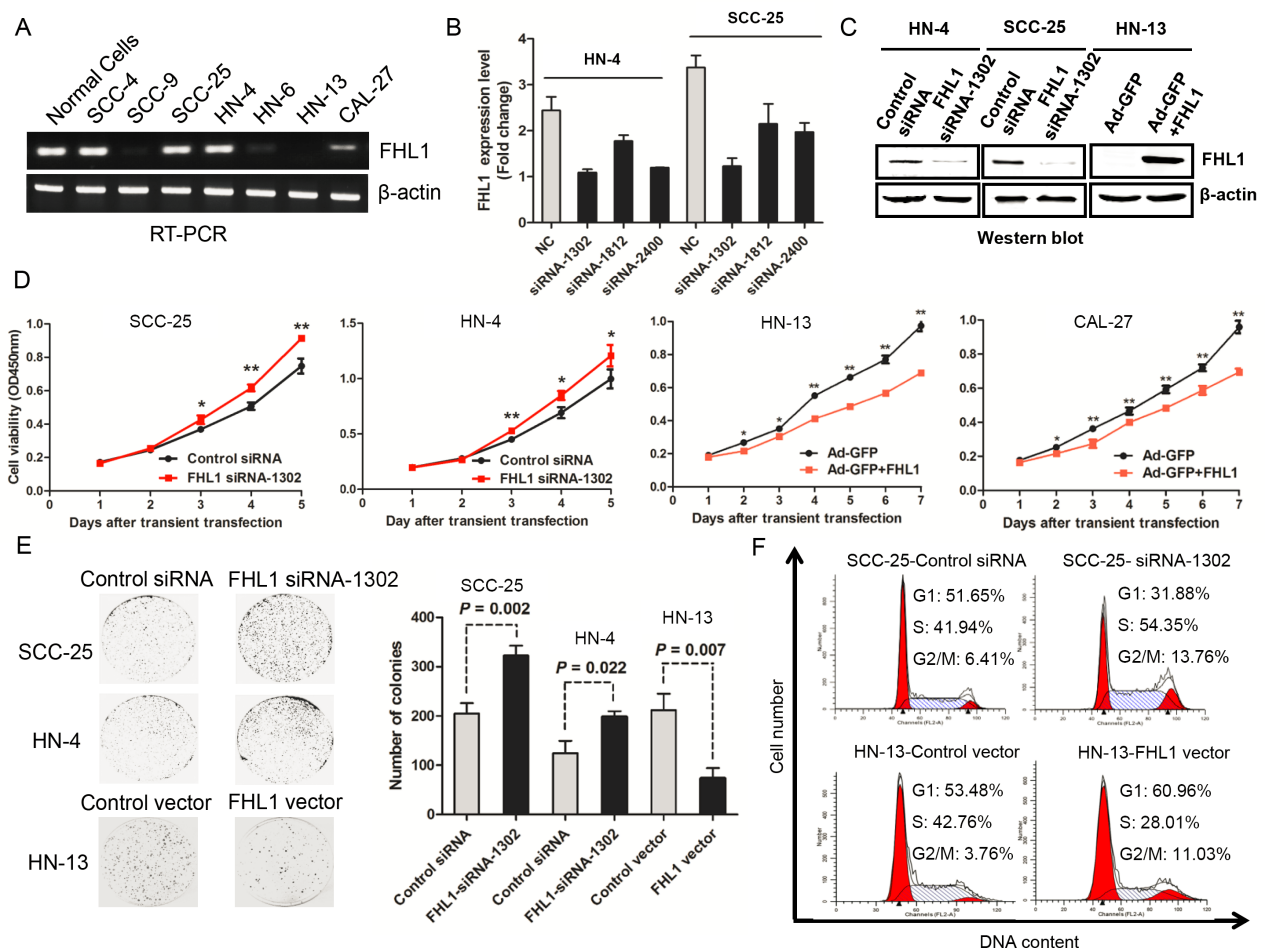
Effects of FHL1 knock down or overexpression on aggressive proliferation of head and neck squamous cell carcinoma (HNSCC) were illustrated (**A**) Expression of FHL1 mRNA level was detected in HNSCC cell lines and normal epithelial cells. (**B**) Silencing effect of FHL1 by three siRNAs was evaluated in HN-4 and SCC-25 cells. (**C**) Expression of FHL1 was detected after FHL1 knock down by siRNA-1302 or FHL1 overexpression by Ad-GFP+FHL1 in HN-4, SCC-25 and HN-13 through using Western blotting assay. (**D**) The effect of FHL1 knockdown by siRNA-1302 or FHL1 overexpression by Ad-GFP+FHL1 on the proliferation of SCC-25, HN-4, HN-13 and CAL-27cells analyzed with the CCK-8 cell-counting kit. (**E**) The effect of FHL1 ablation by siRNA-1302 or FHL1 overexpression by Ad-GFP+FHL1 on the colony formation potential of SCC-25, HN-4 and HN-13 cells. (**F**) The distribution of cell cycle after infecting by siRNA-1302 or FHL1 overexpression by Ad-GFP+FHL1 was observed in SCC-25 and HN-13 cells through cell cycle analysis.

In the training cohort (*n* = 105), our results showed that low *FHL1* mRNA levels were significantly associated with poorer tumor differentiation (*p* = 0.020) (Table 1) and worse OS (*p* = 0.018) or DFS (*p* = 0.038) (Figure 1G and 1H). There were no significant associations between *FHL1* mRNA levels and other parameters. In multivariate COX proportional analyses (Supplementary Table S3), *FHL1* mRNA expression status was identified as independent predictors of OS (*p* = 0.036; HR 0.520; Cl, 0.283–0.958) and DFS (*p* = 0.041; HR 0.527; Cl, 0.284– 0.975) in HNSCC patients. In the validation cohort (*n* = 101), a constant decreased FHL1 expression was observed from well-differentiated to poorly differentiated HNSCCs (*p* = 0.025) (Figure 2A). FHL1 expression levels were evaluated by the digital image analysis (IOD value) (Figure 2B). We also observed that FHL1 expressions were strongly associated with poorer OS (*p* = 0.004) and DFS (*p* = 0.005) (Figure 3C and 3D). Additionally, positive correlation between FHL1 protein levels and p16 expression pattern was found in HNSCC patients (*p* = 0.045) (Supplementary Table S2). Multivariate COX proportional analysis revealed that FHL1 expressions were verified as independent predictors of OS (*p* = 0.021; HR 0.404; Cl, 0.187–0.871) and DFS (*p* = 0.011; HR 0.407; Cl, 0.203–0.815) in patients with HNSCCs (Supplementary Table S3).

**Table 1:** Associations between FHL1 mRNA levels and clinical parameters in a training cohort (n = 105)

Characteristic	No. of Patients (%)	FHL1 mRNA expression (2^−ΔCt^ Mean ± SD)	*P* value
Age, y	105 (100)		
≥ 60	55 (52.4)	0.090 ± 0.128	0.197
< 60	50 (47.6)	0.043 ± 0.006	
Tumor status	105 (100)		
Primary	86 (81.9)	0.030 ± 0.077	0.391
Recurrent	19 (18.1)	0.015 ± 0.015	
Sex	105 (100)		
Men	67 (63.8)	0.034 ± 0.086	0.079
Women	38 (36.2)	0.015 ± 0.018	
Smoking history	105 (100)		
Smoker	38 (36.2)	0.026 ± 0.060	0.876
Nonsmoker	67 (63.8)	0.028 ± 0.075	
Alcohol history	105 (100)		
Drinker	29 (27.6)	0.036 ± 0.101	0.436
Nondrinker	76 (72.4)	0.024 ± 0.054	
Tumor size	105 (100)		
≥ 2 cm	91 (86.7)	0.066 ± 0.007	0.277
< 2 cm	10 (9.5)	0.052 ± 0.106	
Unknown	4 (3.8)		
Tumor grade	105 (100)		
I–II	85 (81.0)	0.031 ± 0.077	0.020
III	16 (15.2)	0.010 ± 0.012	
Unknown	4 (3.8)		
TNM stage	105 (100)		
I–II	31 (29.5)	0.054 ± 0.121	0.105
III–IV	69 (65.7)	0.017 ± 0.023	
Unknown	5 (4.8)		
Disease site	105 (100)		
Oral cavity	90 (85.7)	0.075 ± 0.008	0.607
Oropharynx	15 (14.3)	0.019 ± 0.015	
Lymph node metastasis	105 (100)		
pN positive	52 (49.5)	0.018 ± 0.025	0.169
pN negative	51 (48.6)	0.037 ± 0.096	
Unknown	2 (1.9)		
Adjuvant treatment	105 (100)		
Yes	10 (9.5)	0.014 ± 0.011	0.546
No	95 (90.5)	0.029 ± 0.073	

Abbreviations: SD, standard deviation; FHL1, four and a half LIM domains 1; TNM stage, tumor lymph node metastasis stage; pN, pathologic lymph node status.

### *FHL1* knockdown enhanced cell proliferation, colony formation, and facilitating G_1_-S transition

In 7 HNSCC cell lines, HN-4 and SCC-25 cells with high endogenous *FHL1* expression (Figure 3A) were chosen for loss-of-function assay *in vitro*. SiRNA-1302, one of three siRNAs against *FHL1*, performed the best silencing effect both in HN-4 and SCC-25 (Figure 3B) and significantly decreased FHL1 protein level in two cell lines (Figure 3C). As expected, silencing of FHL1 significantly promoted the growth of HN-4 and SCC-25 cells (*p* < 0.05; Figure 3D). Silencing of *FHL1* significantly increased the number of larger colonies (*p* = 0.002 for SCC-25 and *p* = 0.022 for HN-4, respectively; Figure 3E) as well as promoted the G_1_-S transition in SCC-25 cells (Figure 3F). These results suggested that FHL1 knockdown disrupted contact inhibition among these cells and may contribute to both tumor oncogenesis and progression.

### *FHL1* inhibited tumorigenicity by cell-cycle related proteins dysregulation *in vitro*

**Figure 4:**
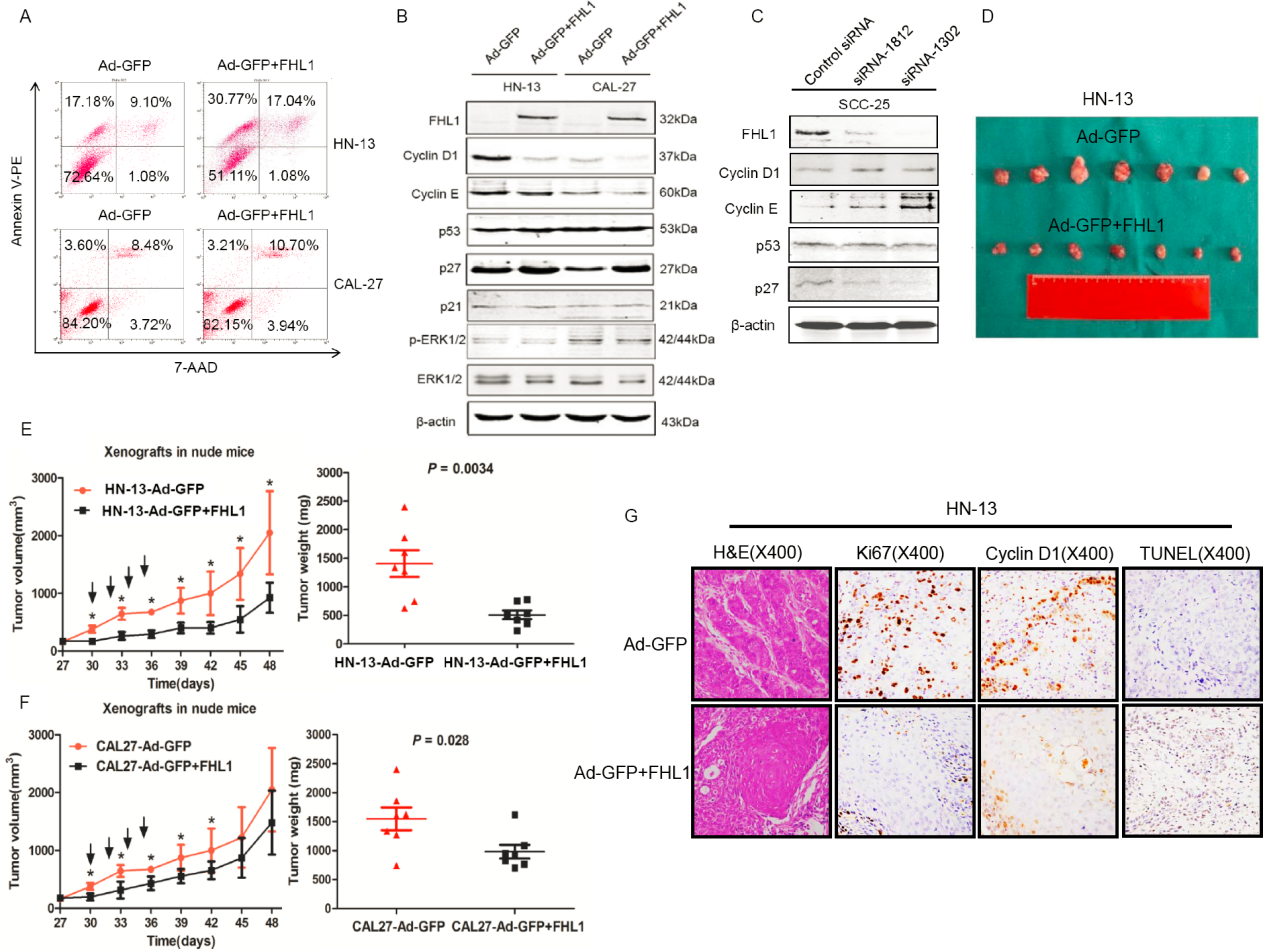
Effects of FHL1 on the apoptosis potential, cell cycle-related proteins and tumorigenicity were evaluated (**A**) The apoptosis potential of FHL1 overexpression with Ad-GFP+FHL1 in HN-13 and CAL-27 cells was examined by 7-AAD and Annexin V-PE double staining through flow cytometry. (**B**) Cell cycle-related proteins were assessed by Western blotting after FHL1 overexpression with Ad-GFP+FHL1 in HN-13 and CAL-27 cells. (**C**) Cell cycle-related proteins were assessed by Western blotting after FHL1 ablation with siRNA-1812 and siRNA-1302 in SCC-25 cells. (**D**) Photographs of the tumors that were surgically removed from mice in each group after they were killed at the end of 7 weeks from the HN-13 cells inoculation. (**E**) The left diagram showed that the time course is from growth of HN-13 xenograft tumors that developed into approximately 100 mm^3^ in mice to the end of 7 weeks after the cell inoculation. Black arrow indicated intratumoral injection with Ad-GFP+FHL1 or Ad-GFP; the right diagram demonstrated that weight of individual tumors surgically removed from the animals in each group. (**F**) The time course curve and weight diagram for CAL-27 xenograft tumors. (**G**) Representative H & E staining and immunohistochemical staining for Ki67, Cyclin D1 as well as TUNEL assay were conducted in HN-13 cell line xenografts each group (original magnification, ×400).

Based on *FHL1* expression pattern in HNSCC cells, we transfected an adenoviral vector containing *FHL1* (Ad-GFP-FHL1) into HN-13 and CAL-27 cells with low FHL1 expression. FHL1 overexpression suppressed the growth and colony formation of these cells (*p* < 0.05; Figure 3D and 3E) as well as caused G_1_-S arrest in HN-13 cells (Figure 3F). To determine whether FHL1 expression induced apoptosis in HNSCC cells, we assessed the fraction with positive staining for 7-amino-actinomycin (7-AAD) and Annexin V-PE in HNSCC cells with FHL1 overexpression and the control cells (47.81% vs 26.28% for HN-13 and 13.91% vs 12.08% for CAL-27; Figure 4A), suggesting that apoptosis-induced inhibitory effect processes a cell-specific manner. Our results also showed that FHL1 overexpression enhanced expression of p27 as well as reduced expression of Cyclin D1 and cyclin E in HN-13 and CAL-27 cells (Figure 4B). Inversely, downregulation of FHL1 decreased expression of p27 as well as promoted expression of Cyclin D1 and Cyclin E in SCC-25 cells (Figure 4C).

### FHL1 overexpression suppressed tumorigenicity *in vivo*

The adenoviral vectors containing *FHL1* were intratumorally injected into xenograft model derived from HN-13 and CAL-27 cells in order to assess whether FHL1 was as a therapeutic target or not. Our findings showed that FHL1 overexpression inhibited tumor growth in both two cell lines (Figure 4D, 4E and 4F). Furthermore, the xenograft tumors with FHL1 overexpression possessed a significantly reduced nuclear Ki67, Cyclin D1 labeling index and enhanced apoptotic potential in HN-13 cell line xenograft (Figure 4G). A notably reduced nuclear Ki-67 and Cyclin D1 labeling index but not enhanced apoptotic potential was found in CAL-27 cell line xenograft with FHL1 overexpression (Supplementary Figure S2). The above findings *in vivo* were consistent with that *in vitro*. Interestingly, these tumors also showed a well differentiated histology pattern whereas the control tumors were poorly/moderately differentiated (Figure 4G and Supplementary Figure S2).

### Genetic and epigenetic alterations of the *FHL1* locus in HNSCCs

**Figure 5:**
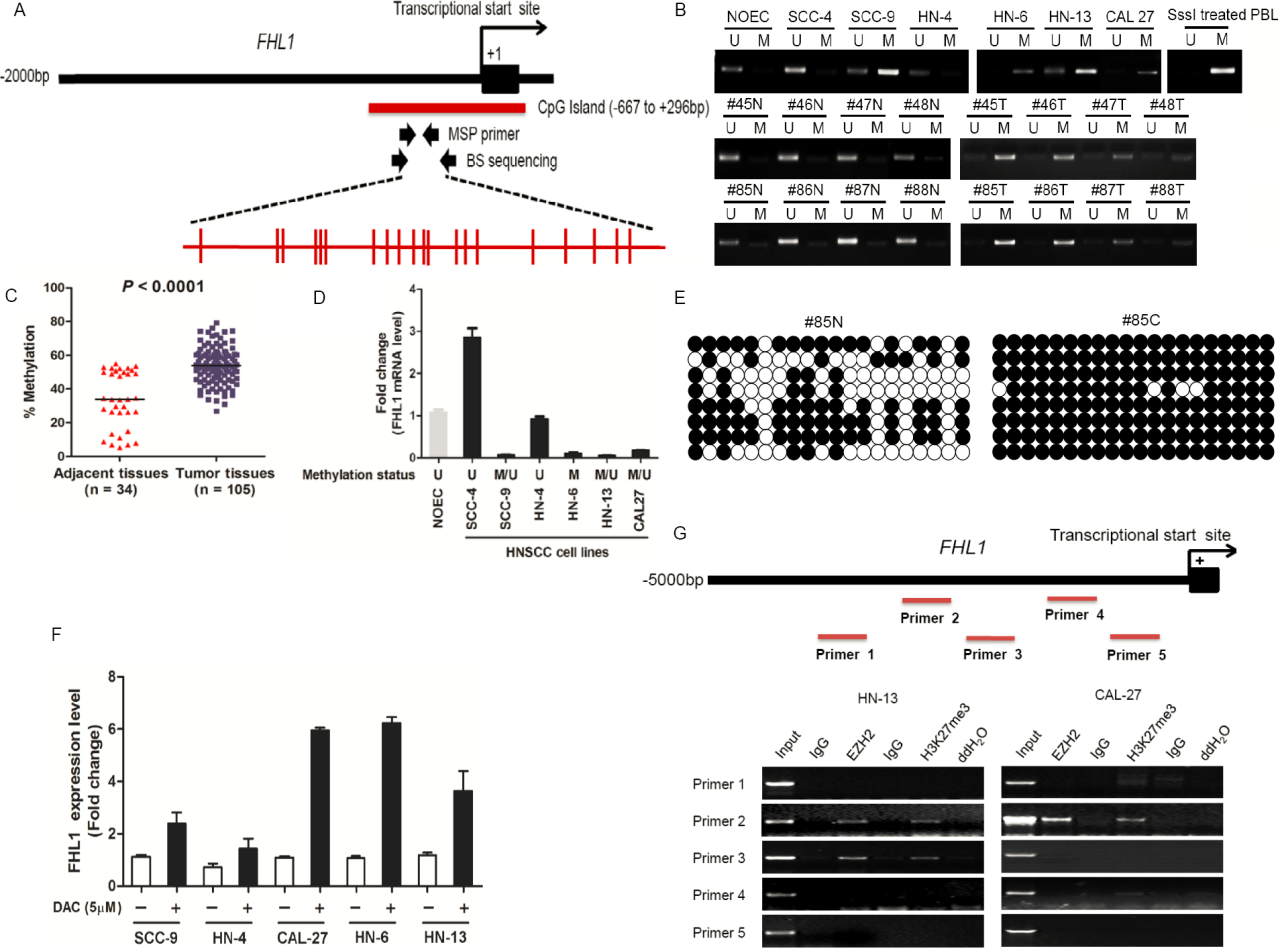
DNA and EZH2-mediated histone hypermethylation together contribute the silencing of *FHL1* in HNSCC (**A**) Schematic representations of the location of CpG island within the promoter of *FHL1* and of the primers designed against the promoter region for Methylation-specific polymerase chain reaction (MSP-PCR) and bisulfite-treated DNA sequencing (BS). Red ticks indicated CpG site in CpG island region. (**B**) Methylation status of *FHL1* was investigated in normal oral epithelial cell (NOEC), HNSCC cell lines and eight representative paired HNSCCs by MSP-PCR. (**C**) Methylation status of *FHL1* was further revealed in paired adjacent tissues (*n* = 34) and HNSCCs (*n* = 105) by MSP-PCR. (**D**) The negative association between *FHL1* mRNA expression and *FHL1* methylation status was illustrated. (**E**) Methylation status of *FHL1* was determined in a representative paired adjacent tissue and HNSCC sample by BS. (**F**) Expression restoration of FHL1 was also observed in five HNSCC cells by real-time PCR after the 5-Aza-dC induction. (**G**) Schematic representations of the location of putative binding region within the promoter of *FHL1* and of the five primers designed against the promoter region for Chromatin Immunoprecipitation (ChIP)-PCR, and notably occupancy of EZH2 and H3k27me3 were found in both HN-13 and CAL-27 cells.

To address whether genetic alteration cause loss-of-function of *FHL1*, we performed the mutational analysis of *FHL1* in its all seven exons in HNSCC cell lines. However, none point mutations were found in HNSCC cell lines (date not shown). We also detected DNA methylation status of *FHL1* by MSP-PCR, BS and 5-Aza-dC induction, respectively. The distribution and localization of CpG sites in *FHL1* promoter were showed in Figure 5A. The result showed that DNA hypermethylation of *FHL1* was found in 4 (66.67%) out of 6 HNSCC cell lines and in 60 (57.14%) out of 105 HNSCCs. The methylation level of the CpG Island in the *FHL1* promoter was significantly higher in HNSCC cells and HNSCCs than that of NOEC and ANT (Figure 5B and 5C). *FHL1* methylation level was inversely correlated with its expression level (Figure 5D). The BS was further validated for the results from MSP-PCR (Figure 5E). *FHL1* gene transcription was reactivated in all five HNSCC cell lines after treatment with 5-Aza-dC (DAC) (Figure 5F). All above data suggested that epigenetic events at the *FHL1* locus could contribute to downregulation of the gene in some HNSCC samples.

In addition to DNA methylation, EZH2 mediated-H3K27Me3 was gradually regarded as one of the main epigenetic event and its functions was independent of promoter DNA methylation [23, 24]. Interestingly, the result demonstrated that *FHL1* expression was increased upon EZH2 ablation in HNSCC cells (Supplementary Figure S1). Secondly, we randomly designed five ChIP primers at the upstream of FHL1 transcriptional start site (Figure 5G). Using ChIP-PCR, we demonstrated that EZH2 occupancy coincides with H3K27me3 at *FHL1* promoters (Figure 5G).

## DISCUSSION

It is well known that genetic and epigenetic mediated the activation of oncogenes and the inactivation of TSGs mainly contributes to cancer initiation and progression [25]. Identification of novel candidate oncogenes and TSGs would serves to well understand hallmarks of cancer and cancer-related network. Chromosome X-linked TSGs are unique because they can be inactivated by a single hit, suggesting they are more easily contributed to carcinogenesis under the same conditions. Here, we conducted a bioinformatics and Venn analysis of integrated four independent data sets for screening novel X-linked TSGs. Total 48 differentiated expression genes were found including *FHL1* downregulation (Supplementary Table S1), indicating that *FHL1* might be a candidate TSG.

FHL1 protein with an N-terminal half LIM domain, followed by four complete LIM domains, belongs to a family of LIM-only proteins that regulate muscle development, structural maintenance, and signaling [26]. Over 25 different FHL1 mutations have been identified in patients with decreasing body myopathy [27]. In a variety of cardiac disorders, FHL1 expression was also significantly up-regulated whereas its expression is down-regulated in certain cancers, suggesting its various physiological and pathological functions. The downregulation was associated with short survival and deep invasion in gastric cancer [28], and with poor differentiation in lung cancers [29]. In this study, we observed downregulation of *FHL1* expression was significantly associated with poorer differentiation (*p* = 0.020), worse OS (*p* = 0.018) and DFS (*p* = 0.038). In Multivariate Cox proportional analyses, *FHL1* expression was identified as independent predictors of OS and DFS in patients with HNSCC.

In validation cohort, FHL1 expression was further evaluated by IHC. To reduce the subjective drawback of IHC semiquantitative analysis, imaging equipment and software were used in this study. The results also showed that FHL1 expression were significantly associated with tumor grade (*p* = 0.025), p16 (*p* = 0.045), poorer OS (*p* = 0.004) and DFS (*p* = 0.005). Multivariate Cox analysis showed that FHL1 expression (*p* = 0.021; HR 0.404, Cl, 0.187–0.871) together with lymph node metastasis (*p* = 0.017; HR 2.334, Cl, 1.167–4.671) was verified as independent predictors of OS and DFS in patients with HNSCC.

FHL1 was identified functionally interacted with oestrogen receptors (ERs), Smad2/3/4, HIF-1, human T-cell leukemia virus type 1 Tax oncoprotein and ZO-1 in various cancer types [30–34], suggesting that its functions as either a tumor suppressor or oncoprotein. Downregulation of FHL1 promoted the growth of human lung cancer cells, hepatoma cells, and breast cancer cells and reduced EMT in breast adenocarcinoma cells. In this work, to verify the function of FHL1, we performed a series of gain-of-function and loss-of-function assays. Our data demonstrated that overexpression of FHL1 inhibited the proliferation, colony formation potential, and caused cell-cycle G1 arrest of HN-13 by apoptosis induction whereas ablating FHL1 promoted the proliferation, the colony formation potential and G1-S transition of HN-4 and SCC-25, suggesting *FHL1* acts as a tumor suppressor in HNSCCs. Furthermore, our findings revealed that FHL1 modulated the proliferation of HNSCC by dysregulated expression of Cyclin D1, Cyclin E1 and p27. Additionally, overexpression of FHL1 dramatically inhibited the volume and the weight of tumors from HN-13 and CAL-27 xenograft models by inducing cell differentiation, decreasing expression of Ki67, Cyclin D1 and promoting apoptosis of HN-13 xenograft tumor cells.

Both genetic and epigenetic events contribute to loss-of-function of TSG in cancer development. To reveal the possibility mechanism about *FHL1* inactivation, we performed mutational analysis and promoter CpG DNA/histone methylation analysis. Although *FHL1* mutations were frequently identified in some muscle disorder diseases [27, 35], none point mutations were observed in HNSCC cell lines. DNA hypermethylation of *FHL1* have been detected in certain cancer types [36, 37], no further findings have been reported yet in HNSCCs. To evaluate DNA methylation status of *FHL1* in HNSCCs, we first used bioinformatics tools to determine a novel CpG island localized in **−**667 bp to + 296 bp of *FHL1* (relative to transcriptional start site as +1). Subsequently, MSP-PCR, BS and 5-Aza-dC induction were employed to measure of promoter methylation status of *FHL1* in HNSCCs. Our data indicated that aberrant DNA hypermethylation contributed to the downregulation of *FHL1* in HNSCCs. Previous study showed that Src utilizes Cas to suppress the expression of the LIM-only protein FHL1 by inducing the methylation of FHL1 promoter region [38]. Thus, hypermethylation of the promoter region of the FHL1 by Src induction warrants further investigation in HNSCCs. In addition to DNA methylation, EZH2-mediated H3K27Me3 also played an important role in silencing of gene and it has been reported that EZH2 was involved in oral cancer development [18, 20]. Interestingly, by using RNA interference, real time-PCR and ChIP-PCR assays, our data demonstrated that *FHL1* was a novel target of EZH2 in HNSCCs.

It is particularly interesting to note that HPV infection has been strongly implicated in oropharyngeal carcinogenesis. However, the incidence of HPV infection was diverse from 15% to 67% in HNSCCs according to different regions and HPV types [39]. In this study, none HPV positive was observed in our Chinese HNSCC patients caused by regional difference and sample size limitation. Therefore, additional studies are necessary to validate these findings by using samples from populations with HPV-positive.

Summarily, in this study, we identified FHL1 as a novel epigenetic silencing target, discovered its function as a tumor suppressor and a novel therapeutic target in HNSCCs. FHL1 expression is an independent predictor for prognosis of patients with HNSCCs.

## MATERIALS AND METHODS

### Venn analysis of published microarray datasets

We augmented the analysis of the data with a Venn analysis of four published microarray data from NCBI GEO datasets (GSE2379, GSE6631, GSE3524 and GSE13601) [10–14] to reliably identify a panel of genes notably downregulated in head and neck squamous cell carcinomas (HNSCCs) compared to adjacent normal tissues (ANT). Software RankProd was applied to analyze for the public microarray datasets, which detected differentially expressed gene under two experimental conditions [15], avoiding complicated experimental variables and ‘lab-effects’ which would occur from direct comparison among heterogeneous datasets, even after the normalization process [16, 17]. Four data sets were crossed by Venn analysis. In current study, genes were selected with evidence of differentially expressed genes in HNSCCs with a False Discovery Rate (FDR) < 0.5 and Fold-change (normal/tumor) ≥ 2 fold (Supplementary Table S1).

### HNSCC specimens

Under the study reviewed and approved by the institutional Ethical Committee of Shanghai Ninth People's Hospital affiliated to Shanghai Jiao Tong University, all subjects signed informed consent documents for participation in this study. Two independent cohorts were incorporated into this study including a training cohort and a validation cohort. The training cohort of 105 consecutive patients who were histologically diagnosed with HNSCCs at the Department of Oral Maxillofacial-Head and Neck Oncology, Shanghai Ninth People's Hospital between November 2008 and June 2011 were collected. All samples were obtained by surgery and cut half to half following by quickly frozen in liquid nitrogen and pathologic diagnosis. The patients with primary or recurrent HNSCCs included 67 men (63.8%) and 38 women (36.2%) with a median follow-up of 41 months (interquartile range, 31 to 46 months) (Table 1). Samples from the validation cohort of 101 consecutive patients were assembled from the database based on histologic diagnosis of primary HNSCCs who received radical surgery in the Department of Oral Maxillofacial-Head and Neck Oncology, Shanghai Ninth People's Hospital from August 1989 to March 1993. There were 53 males (52.5%) and 48 females (47.5%) with a median age of 54 years, and their clinicopathological parameters were summarized in Supplementary Table S2. The median follow-up of the patients was 84 months. In this study, tumor from each patient was stained with hematoxylin and eosin (H & E) and staged according to the International Union Against Cancer tumor-lymph node-metastasis (TNM) classification system.

### Cell lines

CAL-27, SCC-4, SCC-9 and SCC-25 cell lines were purchased from the ATCC and maintained according to the ATCC recommendations. WSU-HN-4, HN-6, HN-13 cell lines and normal epithelial cells (NECs) were acquired and cultured as well as genetic identified as described in the previous study [18, 19].

### Extraction of DNA and RNA

Genomic DNA was extracted from HNSCC cell lines and human HNSCC samples using the DNeasy Tissue kit (Qiagen) according to the manufacturer's protocols. RNA was extracted using TRIZOL solution (Invitrogen) according to the protocols recommended by the manufacturer. The total DNA and RNA concentration and quantity were assessed by absorbance at 260 nm, using a NanoDrop 2000 analyzer (ThermoFisher Scientific)

### RT-PCR

Using a reverse transcription kit (Promega, Madison, USA), 2 μg of total RNA was reverse-transcribed directly to cDNA following the manufacturer's instructions in a total volume of 25 μl. The primer sequences used were as follows: FHL1 forward, 5′-GAC TGG AAG CTT CTT CCC TAA AG-3′; FHL1 reverse, 5′-CCA GCT TCT TAG AGC AGG TAA CA-3′; β-actin forward, 5′-TCA CCC ACA CTG TGC CCA TCT ACG A-3′; and β-actin reverse, 5′-GGG ATG ACT TGT GTT GGA AAA T-3′. Each primer was added at a final concentration of 0.5 μM to a 15 μl reaction mixture in PCR buffer containing 1 μl of cDNA, 0.25 mM of each dNTP, 1.5 mM of MgCl2, and 2.5 units of Taq DNA polymerase. An initial denaturation was performed for 5 minutes at 94°C, and 35 cycles were performed with the following PCR program: denaturing at 94°C for 30 seconds, annealing at 55°C for 30 seconds for FHL1 and 55°C for 30 seconds for β-actin, and elongation at 72°C for 30 seconds. The program was completed with a final extension at 72°C for 5 minutes. Ethidium bromide-stained bands were visualized using UV transillumination, and fluorescence intensity was quantified using the FR- 200 system (FuRi, Shanghai, China). The data from semi-quantitative PCR reactions were normalized against the expression of β-actin from three independent experiments ± the standard deviation (SD). All RT-PCR data were from at least three independent experiments.

### Quantitative real-time PCR assay

All qRT-PCR reactions were performed using an ABI 7300 real-time PCR system (Applied Biosystems corp., Carlsbad, CA) and the SYBR Premix Ex Taq^™^ reagent kit (Takara, Japan). The real-time PCR was performed in a final volume of 15 μl with 1.5 μl of template cDNA at a concentration of 20 ng/μl with 7.5 μl SYBR green I fluorescent dye and 10 pM of each primer for the target gene and β-actin. The primer sequences were sense 5′-TTG TTG GCG GAA GCG TGT AAA ATC-3′ and anti-sense 5′-TCC CTA GTC CCG CGC AAT GAG C-3′ for EZH2, sense 5′-GAC TGG AAG CTT CTT CCC TAA AG-3′ and anti-sense 5′-CCA GCT TCT TAG AGC AGG TAA CA-3′ for FHL1, sense 5′-CCG CCG CGA GTG AGG GTT TT-3′ and anti-sense 5′-CGC TGC CCA TCA TCA TGA CCT GG-3′ for CDKN2A and sense 5′-CCT GGC ACC CAG CAC AAT-3′ and antisense 5′-GGG CCG GAC TCG TCA TAC T-3′ for β-actin. The results of real-time PCR were represented as Ct values, where Ct was a fraction defined as the cycle number at which the sample's fluorescent signal passes a given threshold above the baseline. ΔCt was the difference in the Ct values derived from the specific genes compared to β-actin. Relative mRNA expression level of target gene normalized to β-actin was represented as 2-ΔCt value in our samples. The significance level was defined as a *p* value < 0.05.

### Western blot analysis

As previously described [20], cells were harvested in RIPA buffer (Sigma Aldrich). Whole cell lysate was separated using SDS-PAGE. Primary antibodies against FHL1 (Santa Cruz Biotechnology, sc-374246, 1:1000 diluted), Cyclin D1, Cyclin E, p53, p27, p21, phosph-ERK1/2, and ERK1/2 (Cell signaling Technology) were used in this study. β-actin antibody was used to normalize protein loading.

### Immunohistochemistry

Formalin-fixed, paraffin-embedded tissue samples were cut into 4-μm tissue sections. The avidin-biotin complex (ABC) technique was performed following the manufacturer's instructions for the Vectastatin Elite ABC kit (Vector Laboratories, Burlingame, CA). Briefly, tissue sections were deparaffinized in xylene, rehydrated in graded ethanol, treated with citrate buffer for heat-induced antigen retrieval, and quenched in hydrogen peroxide. Tissue sections were blocked with 2.5% normal serum, incubated overnight at 4°C with anti-FHL1 monoclonal antibody, (Santa Cruz Biotechnology, Dallas, TX, 1:100 diluted) and anti- p16/INK4a monoclonal antibody (1:250), (Clone EPR1473, Epitomics Inc., Burlingame, CA) followed by incubation with biotinylated secondary antibody and then ABC reagent. Diaminobenzidine was used as chromogen, and sections were counterstained with Mayer's hematoxylin (Sigma-Aldrich corp., St Louis, Mo). FHL1 and p16 expression were quantified by computer-based integrated optical density (IOD). For computer-based integrated optical density, Image Pro-Plus (IPP) software was performed in this study and the results were compared with visual assessment. Of the 101 patients with primary HNSCCs, two cases were excluded by the pathologists due to an insufficient number of tumor cells in the sections for evaluation. For FHL1 staining, the cutoff value was 7226982 (IOD). Thus a value greater than or equal to 7226982 (IOD) was considered high expression, whereas a value less than 7226982 (IOD) was considered low expression. For p16 staining, the cutoff value was 5326482 (IOD), thus a value greater than or equal to 5326482 (IOD) was considered high expression, whereas a value less than 5326482 (IOD) was considered low expression.

### FHL1 adenoviral vector construction

Full-length FHL1 ORF (nt261-1103; GeneBank accession number NM_001159700.1) cloned to pEGFP-N1 plasmid were purchased from Genechem Corporation (Shanghai, China). To construct FHL1 recombinant adenovirus vector, full-length FHL1 ORF was inserted into the multiple cloning site of pShuttle-IRES-hrGFP-1 (Stratagene), a shuttle vector that contained a CMV promoter with a GFP. Then, pShuttle-IRES-hrGFP-1-FHL1 and pAdEasy-1(Stratagene) were homologously recombined in E.coli BJ5183. The novel recombined plasmid, Ad-GFP + FHL1 was verified by restriction endonuclease digestions and sequencing. Ad-GFP + FHL1 was propagated in HEK293 cells and a viral stock was harvested from those cells. After 6 cycles of freezing and thawing, cell debris was removed by subjecting the lysed cells to 12,000 g centrifugation. The virus stock was stored at **−**80°C.

### Small interfering RNA

Three siRNAs against FHL1 were designed by BLOCK-iT TM RNAi Designer online software (http://rnaidesigner.invitrogen.com/rnaiexpress/) and chemically synthesized (Shanghai Genepharma Co.) for targeting different coding regions of the gene as follows: siRNA-1302 (5′-CCCUGCAGCAAAGUGAAUUdUdC-3′ and 5′-AAU UCACUUUGCUGCAGGGdUdU-3′) for nt 1302-1324 of FHL1, siRNA-1812 (5′-GCCUGUUUCAGAGGAACAUd CdG-3′ and 5′-AUGUUCCUCUGAAACAGGCdUdC-3′) for nt 1812-1834 of FHL1. Additionally, specific anti-EZH2 siRNA were purchased from Ambion Inc. (Austin, Tex) and control siRNA (5′-UUCUCCGAACGUGUCACGU dTdT-3′ and 5′-ACGUGACACGUUCGGAGAAdTdT-3′) was also synthesized.

### Cell proliferation assay

A cell-proliferation assay was performed to analyze the proliferation potential of transiently transfected FHL1 siRNA compared with negative control siRNA in SCC-25 and HN-4 cells as well as proliferation potential of transiently transfected FHL1 expression vector compared with empty vector in HN-13. Briefly, *In vitro* transient transfection was performed using Lipofectamine 2000 (Invitrogen, Carlsbad, Calif) following the manufacturer's protocol. The cells were harvested and plated in 96-well plates at 1 × 10^3^ cells per well and maintained at 37°C in a humidified incubator. At the indicated time points, 10 μL of the CCK-8 solution were added into the triplicate wells and incubated for 1 hour, and the absorbance at 450 nm was measured to calculate the number of vital cells in each well. Measurements were performed in triplicate, and the mean (± standard deviation) optical density was reported.

### Colony formation

To assay the effect of FHL1 on colony formation, recombinant pEGFP-N1 vectors containing the FHL1 or siRNA against FHL1were transfected into targeted HNSCC cells (empty vector or control siRNA as a control) in 35-mm dishes by Lipofectamine2000 (Invitrogen) for 24 hours, and then stripped and plated onto 100-mm tissue culture dishes. After 2 weeks of selection, the remaining colonies were washed twice with PBS, stained with crystal violet, and counted on crystal violet–stained dishes. All experiments were independently repeated at least 3 times.

### Cell cycle analysis

Targeted HNSCC cells transfected with recombinant pEGFP-N1 vectors containing the FHL1 or siRNA against FHL1 (empty vector or control siRNA as a control) were harvested, fixed in 70% ethanol, and suspended in PI/RNase staining buffer (BD Pharmingen) containing 0.1% sodium citrate and 0.1% Triton X-100. Data analysis was done using FlowJo software.

### Apoptosis assay

The cells infected with the Ad-GFP or Ad- GFP+FHL1 virus were harvested at 72 hours. These cells were then quantified by flow cytometry using the Annexin V-PE Apoptosis Detection Kit (BD Biosciences, USA) according to the manufacturer's protocols. Briefly, trypsinized adherent cells and floating cells were harvested, washed twice with cold PBS and resuspended in 1 × Binding Buffer at a concentration of 1 × 10^6^ cells/ml. Then, 5 μl of Annexin V-PE and 5 μl of 7-AAD were added, and the cells were incubated for 15 minutes at 25°C in the dark. The cells were then resuspended in 400 μl of 1× Binding Buffer and analyzed immediately by BD LSR II flow cytometry (BD Biosciences, USA).

### Tumor formation in nude mice

A total of 2 × 10^6^ HN-13 or CAL-27 cells were injected subcutaneously into the right flank or both flanks of nude mice. Treatment was initiated when tumors reached approximately 100 mm^3^. Ad-GFP+FHL1 and Ad-GFP regents were injected into tumors by intratumoral injection way every other day, respectively. A total of injections four times were performed in this process. Growth curves were plotted based on mean tumor volume within each experimental group at the indicated time points. The tumor dimensions and nude mice weights were measured every 3 days using a digital caliper, and the tumor volume calculated using the following formula: V = π/6 × (larger diameter) × (smaller diameter)^2^. Tumor growth was observed for at least at 3 weeks after the initial treatment. The tumorigenic experiments *in vivo* were performed with 7 mice in each treatment group.

### Mutational analysis of FHL1 in HNSCC cell lines

All seven exons of *FHL1* were amplified in WSU-HN-4, HN-6, HN-13, CAL-27, SCC-4, SCC-9, SCC-25 and NECs using 100 ng of genomic DNA by PCR as indicated in previous study [21]. The PCR products were directly cycle-sequenced with an ABI PRISM 377 automated DNA sequencer.

### MSP-PCR and bisulfite-treated DNA sequencing

As formerly described [22], genomic DNA (1 μg) was denatured by incubation with 0.2 M NaOH. Aliquots of 10 mM hydroquinone and 3 M sodium bisulfite (pH 5.0) were added and the solution was incubated at 50°C for 16 hours. Methylation-specific polymerase chain reaction (MSP-PCR) and bisulfite-treated DNA sequencing (BS) were performed and specific primers for MSP or BS were summarized in Supplementary Table S4. The PCR product was subcloned into a pMD 18-T vector (TaKaRa Inc.) for DNA sequencing on an ABI 3730 sequencer.

### Induction of gene expression by 5-Aza-dC

To induce demethylation of promoter prior to evaluation for induction of FHL1 expression, five HNSCC cell lines were treated with 5 μM 5-Aza-dC (DAC), which was the DNA demethylation reagent for 72 hours.

### ChIP assay

HN-13 and CAL-27 cells with low endogenous FHL1 were selected for ChIP-PCR analysis. For each ChIP assay, antibodies (2 μg) used for ChIP included monoclonal anti-EZH2 (Millipore), polyclonal anti-H3K27Me3 (Upstate) antibody or IgG control (Millipore). The primer sets are listed in the Supplementary Table S4. ChIP enriched DNA and input DNA were subjected to PCR analysis.

### Statistical analysis

For IHC analysis, the associations between FHL1 expression level by IOD and patient characteristics were evaluated using Fisher Exact test for categorical variables and Kruskal-Wallis test for continuous variables. For real-time PCR analysis, the associations between *FHL1* mRNA level and patient characteristics were evaluated using the Kruskal-Wallis test. The log-rank test was used for univariate associations between FHL1 expression level and OS and DFS. Then, all potential prognostic factors with a *p* value < 0.05 from the univariate analysis were incorporated in multivariate analyses. The hazard ratios [HR] with corresponding 95% confidence intervals [CI] and *P* values were reported. Paired *t* test was used for analysis of the *in vitro* and *in vivo* studies. All the analyses were conducted using the SPSS software program (SPSS Standard version 13.0). All tests were two-sided, and *p* values < 0.05 were considered statistically significant.

## SUPPLEMENTARY MATERIALS FIGURES AND TABLES




