# Supplementary material for: X-linked FHL1 as a novel therapeutic target for head and neck squamous cell carcinoma
Source: Oncotarget. 2016 Feb 18;7(12):14537–50. doi: 10.18632/oncotarget.7478 (PMC4924734; doi:10.18632/oncotarget.7478)
Supplement: Supplementary file 1 [file oncotarget-07-14537-s001.pdf]

X-linked *FHL1* as a novel therapeutic target for head and neck squamous cell carcinoma

Supplementary Materials

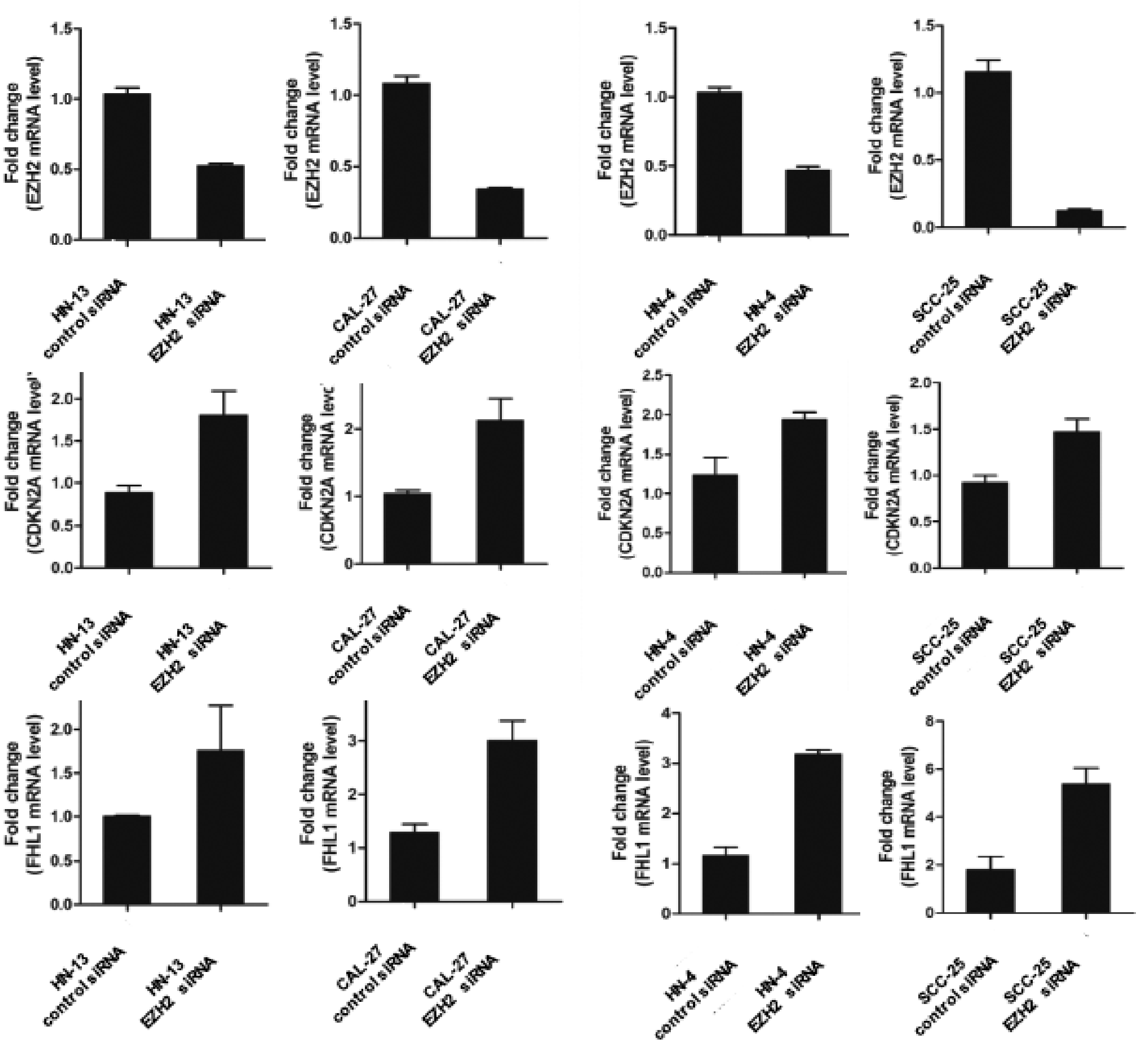

Supplementary Figure S1: Expression restoration of FHL1 and CDKN2A was also observed in HN-13 and CAL-27 cells by real-time PCR after EZH2 knock down.

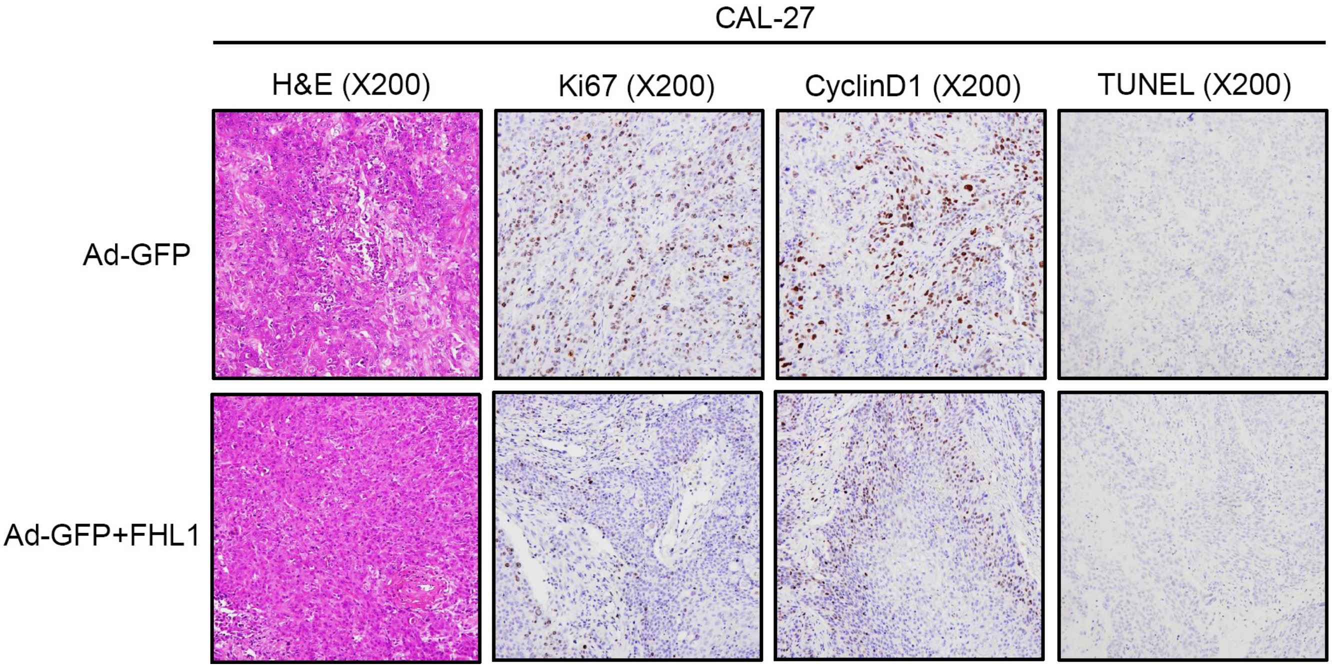

Supplementary Figure S2: Representative H & E staining and immunohistochemical staining for Ki67, Cyclin D1 as well as TUNEL assay were conducted in CAL-27 cell xenografts each group (original magnification, ×200).

Supplementary Table S1: Differentially expressed genes in HNSCCs distributed within four GEO datasets

Supplementary Table S2: Associations between FHL1 protein levels and clinical parameters in the validation cohort (*n* = 101)

		No. of Patients (%) FHL1 Expression, *n* = 99[a]		
		IOD Value		
**Characteristic**	**Total *n* = 101**	**< Median Negative**	**≥ Median Positive**	***P* value**
Age, y				
Mean ± SD	54.3 ± 11.8	54.5 ± 12.0	54.5 ± 11.6	0.685
Median	54	54	55	
Range	28–75	28–75	32–74	
Sex				
Men	53 (52.5)	27 (27.3)	25 (25.3)	0.548
Women	48 (47.5)	21 (21.2)	26 (26.3)	
Smoking history				
Smoker	28 (27.7)	13 (13.1)	14 (14.1)	0.755
Nonsmoker	66 (65.3)	32 (32.3)	33 (33.3)	
Ever	4 (4)	3 (3)	1 (1)	
Unknown	3 (3)	0 (0)	3 (3)	
Alcohol history				
Drinker	16 (15.8)	10 (10.1)	5 (5.1)	0.199
Nondrinker	75 (74.3)	33 (33.3)	41 (41.5)	
Ever	6 (5.9)	4 (4)	2 (2)	
Unknown	4 (4)	1 (1)	3 (3)	
Tumor grade				
I–II	90 (89.1)	39(39.4)	49 (49.5)	0.025
III	11 (10.9)	9(9.1)	2 (2)	
TNM stage				
I–II	46 (45.5)	20 (20.2)	26 (26.2)	
III–IV	50 (50.5)	25 (25.3)	25 (25.3)	0.546
Unknown	5 (5)	3 (3)	2 (2)	
Disease site				
Oral cavity	95 (94.1)	47 (47.5)	46(46.5)	0.206
Oropharynx	6 (5.9)	1 (1)	5(5)	
Lymph node metastasis				
pN positive	27 (27.3)	14 (14.1)	13 (13.1)	0.650
pN negative	69 (69.7)	31 (31.3)	38 (38.5)	
Unknown	5 (5)	3 (3)	2 (2)	
p16[a]				
Positive	52 (52.5)	20 (20.2)	32 (32.3)	0.045
Negative	47 (47.5)	28 (28.3)	19 (19.2)	

Abbreviations: FHL1, four and a half LIM domains 1; IRS: immunoreactive score; IOD: integrated optical density; SD, standard deviation; pN, pathologic lymph node status; TNM stage, tumor lymph node metastasis stage.

[a]Expression levels of FHL1 or p16 were unavailable in 2 patients due to the lack of tumor cells for evaluation.

Supplementary Table S3: Univariate and multivariate Cox proportional hazards regression models for estimating OS and DFS of patients in the training cohort (n = 105) and the validation cohort (n = 101)

Characteristics	Training cohort (n = 105)			Validation cohort (n = 101)		
	HR	95% Cl	P value	HR	95% Cl	P value
**Overall survival**						
Univariate analysis						
Age (≥ 60 y vs < 60 y)	1.762	0.963 to 3.223	0.066	0.510	0.243 to 1.072	0.075
Sex (men vs women)	1.079	0.585 to 1.989	0.808	1.703	0.838 to 3.464	0.141
Tumor status (primary vs recurrent)	1.898	0.961 to 3.751	0.065	N/A	N/A	N/A
Smoking history (smoker vs nonsmoker)	0.889	0.478 to 1.653	0.710	1.187	0.676 to 2.084	0.551
Alcohol history (drinker vs nondrinker)	0.993	0.513 to 1.923	0.984	1.134	0.646 to 1.990	0.662
Tumor size (≥ 2 cm vs < 2 cm)	1.284	0.457 to 3.609	0.635	N/A	N/A	N/A
TNM stage (I–II vs III–IV)	2.112	1.065 to 4.190	0.032	2.493	1.186 to 5.244	0.016
Tumor grade (I–II vs III)	2.090	1.029 to 4.244	0.042	1.612	1.060 to 2.453	0.026
Disease site (oral cavity vs oropharynx)	0.665	0.262 to 1.687	0.390	0.926	0.221 to 3.868	0.916
pN (positive vs negative)	2.056	1.106 to 3.823	0.023	2.648	1.332 to 5.264	0.005
Adjuvant treatment (yes vs no)	0.753	0.232 to 2.432	0.636	N/A	N/A	N/A
FHL1 expression (high vs low)	0.494	0.271 to 0.901	0.022	0.357	0.170 to 0.751	0.007
Multivariate analysis						
FHL1 expression (high vs low)	0.520	0.283 to 0.958	0.036	0.404	0.187 to 0.871	0.021
TNM stage (I–II vs III–IVA)	1.525	0.711 to 3.273	0.279	1.793	0.706 to 4.553	0.220
Tumor grade (I–II vs III)	1.435	0.689 to 2.990	0.335	1.368	0.885 to 2.113	0.158
pN (positive vs negative)	1.723	0.876 to 3.388	0.115	2.334	1.167 to 4.671	0.017
**Disease-free survival**						
Univariate analysis						
Age (≥ 60 y vs < 60 y)	1.887	1.030 to 3.456	0.054	0.473	0.234 to 0.958	0.068
Sex (men vs women)	1.185	0.643 to 2.185	0.586	1.352	0.701 to 2.607	0.368
Tumor status (primary vs recurrent)	1.829	0.925 to 3.616	0.082	N/A	N/A	N/A
Smoking history (smoker vs nonsmoker)	1.012	0.544 to 1.883	0.969	1.093	0.635 to 1.882	0.748
Alcohol history (drinker vs nondrinker)	1.106	0.571 to 2.142	0.766	1.087	0.635 to 1.860	0.761
Tumor size (≥ 2 cm vs < 2 cm)	1.225	0.436 to 3.441	0.700	N/A	N/A	N/A
TNM stage (I–II vs III–IV)	2.003	1.011 to 3.966	0.046	2.341	1.175 to 4.664	0.016
Tumor grade (I–II vs III)	1.885	0.930 to 3.820	0.079	1.477	0.977 to 2.233	0.064
Disease site (oral cavity vs oropharynx)	0.637	0.215 to 1.616	0.343	1.314	0.403 to 4.280	0.651
pN (positive vs negative)	2.077	1.117 to 3.863	0.021	2.959	1.547 to 5.662	0.001
Adjuvant treatment (yes vs no)	0.671	0.208 to 2.165	0.504	N/A	N/A	N/A
FHL1 expression (high vs low)	0.539	0.296 to 0.983	0.044	0.390	0.196 to 0.777	0.007
Multivariate analysis						
FHL1 expression (high vs low)	0.527	0.284 to 0.975	0.041	0.407	0.203 to 0.815	0.011
TNM stage (I–II vs III–IVA)	1.831	0.817 to 4.103	0.142	1.433	0.585 to 3.508	0.431
pN (positive vs negative)	1.648	0.838 to 3.242	0.148	2.067	0.888 to 4.812	0.092

Abbreviations: CI, confidence interval; HR, hazard ratio; TNM, tumor-lymph node-metastasis classification; pN, pathologic lymph node status; FHL1, four and a half LIM domains 1; N/A, not applicable.

Supplementary Table S4: Primer list for MSP, BS and ChIP-PCR

Name	Sequence
MSP-FHL1-M-Forward	5′- GTAAGTTATCGGGTTTCGAAGTC-3′
MSP-FHL1-M-Reversal	5′- ACAACCAAATAAAAATAACGTCTCG-3′
MSP-FHL1-U-Forward	5′- TGTAAGTTATTGGGTTTTGAAGTTG-3′
MSP-FHL1-U-Reversal	5′- AACCAAATAAAAATAACATCTCACC-3′
BS-FHL1-Forward	5′- GCT CTGGATGGTGGCACTGGG -3′
BS-FHL1-Reversal	5’- AAAGACAGCCAAGTGAGGGTGG -3′
ChIP- FHL1-Primer1-Forward	5′-GCTGGGGAAAACAGACACAT-3′
ChIP- FHL1-Primer1-Reversal	5′-TCACGTGTCCTGTCAATGGT-3′
ChIP- FHL1-Primer2-Forward	5′-AAAGAAGCAGCCACTTTGGA-3′
ChIP- FHL1-Primer2-Reversal	5′-TCTGGACAGAGCACCCTCTT-3′
ChIP- FHL1-Primer3-Forward	5′- TGTTCAATGCCTTTCCATGA-3′
ChIP- FHL1-Primer3-Reversal	5′-AGCGAGTAGGGACAAGCAAA-3′
ChIP- FHL1-Primer4-Forward	5′-TGGTGAAAGACTTGGGGAAC-3′
ChIP- FHL1-Primer4-Reversal	5′-GGAAAGCTGGGGAGAGTACC-3′
ChIP- FHL1-Primer5-Forward	5′-GCTTTTTCCTGGCTCTTTCA-3′
ChIP- FHL1-Primer5-Reversal	5′-GAGGTGGGAGCAACAAAGAC-3′

Abbreviations: MSP, methylation specific PCR; BS, bisulfite sequencing; M, methylation; U, unmethylation; ChIP, chromatin immunoprecipitation.
